# V/P SPECT as a diagnostic tool for pregnant women with suspected pulmonary embolism

**DOI:** 10.1007/s00259-015-3056-z

**Published:** 2015-04-28

**Authors:** Marika Bajc, Berit Olsson, Anders Gottsäter, Cecilia Hindorf, Jonas Jögi

**Affiliations:** Clinical Physiology and Nuclear Medicine, Skåne University Hospital and Lund University , 221 85 Lund, Sweden; Radiation Physics, Skåne University Hospital, Lund, Sweden; Vascular Diseases, Skåne University Hospital, Malmö, Sweden

**Keywords:** Pregnancy, Pulmonary embolism, V/P SPECT, Radiation exposure

## Abstract

**Purpose:**

The purpose of the study was to assess the prevalence of pulmonary embolism (PE) and other lung diseases among pregnant women with suspected PE and to calculate the radiation exposure to patient and fetus in this population. As a secondary aim, we evaluated the negative predictive value of a normal ventilation/perfusion single photon emission computed tomography (V/P SPECT) examination in pregnancy.

**Methods:**

We studied all 127 pregnant women who had suspected PE and had undergone V/P SPECT at our institution in the course of a 5-year period. Radiation exposure to patient and fetus and the negative predictive value of a normal V/P SPECT examination were also measured.

**Results:**

V/P SPECT identified PE in 11 women (9 %). Moreover, in 15 women (12 %) the examination revealed pneumonia (in 2 cases in addition to PE) and in 1 woman signs of airway obstruction were revealed. Among the 116/127 women (91 %) where PE was ruled out by V/P SPECT, none was diagnosed subsequently with PE or deep venous thrombosis (DVT) during the same pregnancy or puerperal period. For P SPECT, the calculated fetal absorbed dose was < 0.6 mGy,and the calculated breast absorbed dose 0.6 mGy. For V SPECT, the calculated fetal absorbed dose was < 0.014 mGy and the breast absorbed dose 0.25 mGy.

**Conclusion:**

The prevalence of PE was low (9 %) among pregnant women with suspected disease. Pneumonia was diagnosed in 12 % of patients. The negative predictive value of V/P SPECT was high, and the radiation exposure from V/P SPECT was low both for fetus and patient.

## Introduction

The risk for pulmonary embolism (PE) is increased about fivefold during pregnancy and the puerperal period [1, 2] due to both changes in the coagulation system and mechanical factors such as vein compression, and more than 50 % of events occur in the first 20 weeks of pregnancy [3]. As clinical symptoms are unspecific, it is important to take into account the possibility of PE when a pregnant woman experiences symptoms such as chest pain, dyspnoea, pyrexia, tachycardia, leg pain and swelling (particularly in the left leg) or lower abdominal pain. However, many of these symptoms may be due to the physiological stress of pregnancy or to other cardiopulmonary diseases, such as pneumonia or on rare occasions left heart failure. In the initial diagnostic evaluation of PE, testing of clinical probability is important, but few of the test algorithms have been evaluated during pregnancy [4]. D-dimer is usually increased during pregnancy, and therefore current evidence does not support the use of negative D-dimer to rule out PE in a pregnant woman [5]. Therefore, a clinical suspicion of PE always needs to be confirmed by an imaging test. Currently, ventilation/perfusion single photon emission computed tomography (V/P SPECT) [6, 7] or planar pulmonary scintigraphy [8] is recommended by different European guidelines as an initial imaging modality, based on its low radiation exposure, high sensitivity and specificity and possibility for follow-up. The American Society of Thoracic Radiology [9] also recommends planar pulmonary scintigraphy as the preferred imaging method during pregnancy.

We studied all pregnant women who underwent V/P SPECT at our institution for suspected PE over a 5-year period. The primary aims of the study were to assess the prevalence of PE and other lung diseases and to calculate the radiation exposure to patient and fetus in this population. As a secondary aim, we evaluated the negative predictive value of a normal V/P SPECT examination in pregnancy.

## Materials and methods

In the course of a 5-year period (2009–2013), 127 pregnant women (mean age 30 years, range 18–48) were referred for examination with V/P SPECT due to suspected PE at Skåne University Hospital, Lund, Sweden. During the study period, 30 patients (24 %) were examined in the first trimester (weeks 1–15), 59 (46 %) in the second trimester (weeks 16–27) and 38 (30 %) in the third trimester (weeks 28–40). In accordance with the European guidelines [6, 7], the 2-day protocol was implemented during the first trimester of pregnancy: P SPECT only on the first day and V SPECT performed the next day only if indicated (Table 1). Chest X-ray was performed only if considered clinically indicated. Patient files from all hospitals in the region were checked for potential later PE or deep venous thrombosis (DVT) diagnoses made in the 127 women during the same pregnancy and puerperal period. Clinical characteristics and symptoms at presentation in the 127 women are shown in Table 2.

**Table 1:** Different examination techniques used in 127 pregnant women undergoing V/P SPECT for suspected PE

Trimester	Patients	P SPECT	V SPECT	V/P SPECT
1st (weeks 1–15)	30	14	13	3
2nd (weeks 16–27)	59	6	1	52
3rd (weeks 28–40)	38			38

Data shown separately for women in each trimester of pregnancy

**Table 2:** Clinical characteristics and symptoms at presentation [*n* (%)] in 127 pregnant women undergoing V/P SPECT for suspected PE

Clinical characteristics	*n* (%)
Previous VTE	6 (5)
Family history of VTE	12 (9)
Pneumonia	7 (6)
Bronchial asthma or chronic obstructive pulmonary disease	8 (6)
Pulmonary histiocytosis	1 (1)
Cystic fibrosis	1 (1)
Pre-eclampsia	1 (1)
Symptoms	
Chest pain	52 (41)
Dyspnoea	69 (54)
Cough	15 (12)
Syncope	3 (2)
High fever	2 (2)
Haemoptysis	6 (5)
Swollen leg	7 (6)

*VTE* venous thromboembolism

### Absorbed dose calculation

Breast doses were calculated using data from ICRP 53 and ICRP 80 [10, 11] and fetal doses were calculated according to Russell et al. [12]. The ^99m^Tc-Technegas was not assumed to cross over to the placenta, which means that the only contribution to the fetal dose is the radiation from activity within the lungs of the mother. The specific absorbed fractions for photons were obtained from www.doseinfo-radar.com, accessed on 23 Apr 2014, and were recalculated to the photon energy for ^99m^Tc (140 keV). The ^99m^Tc-Technegas was assumed to remain within the lungs until complete decay as no biological excretion of the radiopharmaceutical occurs.

### Perfusion (P) SPECT

P SPECT was performed in accordance with the guidelines of the European Association for Nuclear Medicine (EANM) [6, 7] with a dual-head gamma camera in the supine position, after i.v. administration of 50 MBq ^99m^Tc-macroaggregated albumin (MAA, TechneScan LyoMAA®, Mallinckrodt Medical, Petten, The Netherlands).

### Ventilation (V) SPECT

V SPECT was performed after inhalation of aerosolized ^99m^Tc-Technegas® (Cyclomedica Ltd, Lucas Heights, NSW, Australia), reaching 30 MBq in the lungs. The procedure was carried out with the patient in the supine position over a period of 11 min (10 s per stop, 64 stop for each camera) as described elsewhere [13].

### V/P SPECT protocol

The 1-day protocol for V/P SPECT started with inhalation of up to 30 MBq ^99m^Tc-Technegas to the lung. V SPECT was performed in the supine position, with acquisition lasting 11 min. Without changing the patient position, P SPECT followed immediately, after i.v. injection of 120 MBq ^99m^Tc-MAA, with acquisition lasting 5 min. The methodology has been described in full elsewhere [13, 14]. Over the same 5-year period, 61 pregnant women with suspected PE were examined with multidetector CT (MDCT), 37 (61 %) of them during the night or weekends when V/P SPECT was not available.

### Statistics and ethics

The study is a descriptive analysis, which had been approved by the local Ethics Committee of Lund University.

## Results

**Fig. 1:**
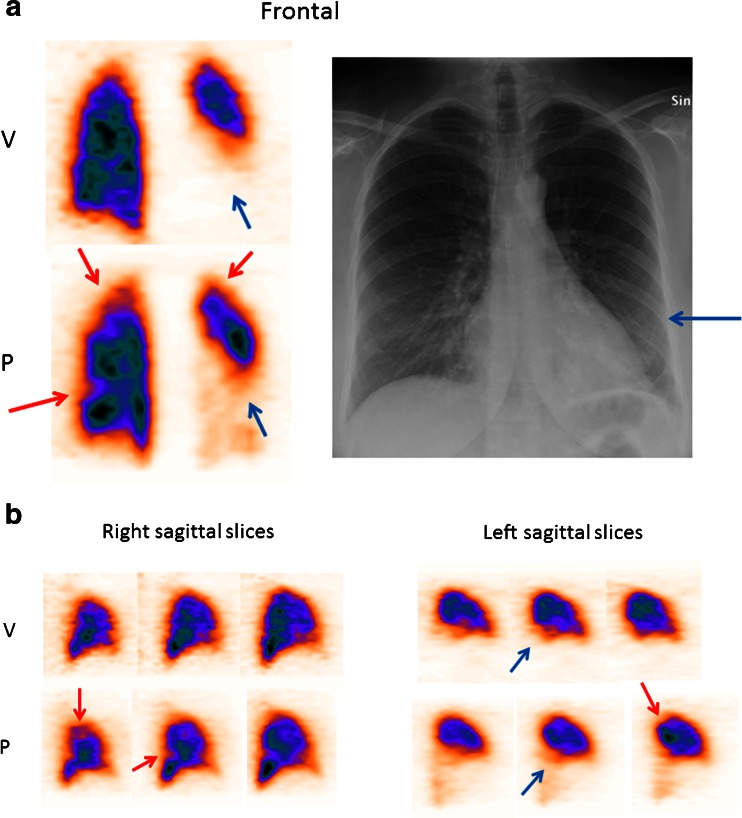
Frontal (**a**) and sagittal (**b**) slices from V/P SPECT and chest X-ray in a pregnant woman diagnosed with both PE and pneumonia in the second trimester of pregnancy. *Red arrows* indicate segmental perfusion defects in areas with preserved ventilation representing PE in the right and left lung. *Blue arrows* indicate absent ventilation and reduced perfusion representing pneumonia in the lower left lung

PE was identified using V/P SPECT in 11 (9 %) of the 127 patients. The extent of PE was small to medium, not exceeding 40 % of the pulmonary vascular bed in any case. Moreover, in 15 patients (12 %) the examination revealed pneumonia [7] (in 2 of these cases in addition to PE), and in 1 patient it indicated signs of airway obstruction, defined in [15] (Table 3). Among the 30 patients examined during the first trimester, PE was diagnosed in 5. In this group, 14 patients underwent P SPECT only and only 1 in this group was diagnosed with PE, whereas 13 patients underwent V SPECT the next day and among them 4 were diagnosed with PE, 1 with concomitant pneumonia. In four patients pneumonia was observed as the sole finding. Among patients examined during the second trimester, 4 of 59 were diagnosed with PE. Six patients underwent P SPECT only and none was positive for PE. One more patient needed a V SPECT the next day which showed findings typical for pneumonia. Among 52 other patients undergoing the 1-day V/P SPECT protocol 4 were diagnosed with PE, 1 with concomitant pneumonia (Fig. 1) that was also clinically treated. Moreover, one woman was diagnosed with airway obstruction.

**Table 3:** Results of P and V SPECT in 127 pregnant women undergoing V/P SPECT for suspected PE

		Trimester		
		1	2	3
PE	11 (9%)	5	4	2
Pneumonia	15 (12%)	4	6	5
Left heart failure	1 (<1%)		1	
Airway obstruction	1 (<1%)		1	

Results shown both in total and separately for women in each trimester of pregnancy

Among those examined during the last trimester, 3 of 38 women were diagnosed with PE, 1 of whom also had pneumonia. In four other women, signs of pneumonia were observed. Among the 116 of 127 patients (91 %) in whom PE was ruled out by V/P SPECT, none was diagnosed with PE or DVT during the same pregnancy or the puerperal period. A negative study was thus 100 % predictive of no clinically apparent emboli in this time period.

Calculated breast absorbed doses were 0.2 mGy after inhalation of 30 MBq of ^99m^Tc-Technegas and 0.6 mGy after intravenous injection of 120 MBq ^99m^Tc-MAA. If only P SPECT was performed calculated breast absorbed dose was 0.25mGy [10–12]. The doses to the fetus were calculated to the stage of gestation (Table 4) [10–12].

**Table 4:** Fetal absorbed dose (mGy) calculated to the stage of gestation after i.v. injection of ^99m^Tc-MAA and inhalation of ^99m^Tc-Technegas in 127 pregnant women undergoing V/P SPECT for suspected PE

Stage of gestation	Absorbed dose after 50 MBq ^99m^Tc-MAA	Absorbed dose after 30 MBq ^99m^Tc-Technegas	Absorbed dose after 120 MBq ^99m^Tc-MAA
Early	0.14	0.007	0.34
3 months	0.20	0.007	0.48
6 months	0.25	0.011	0.60
9 months	0.20	0.014	0.48

### Multidetector computed tomography

Among pregnant women undergoing MDCT during the 5-year period, 3 of 61 showed signs of PE. In two of these, the examination was not considered as optimal. However, six patients showed parenchymal changes, and in six other cases the examination was reported by the radiologist as inconclusive and not allowing exclusion of PE. One patient underwent two MDCT examinations that were both considered inconclusive. Among 127 pregnant women, 7 were suspected for DVT. Five were examined with ultrasound, and only one was pathological.

## Discussion

This is the first study reporting the value of V/P SPECT during pregnancy. It established that we were able to follow the European guidelines [6, 7] and use the proposed 2-day protocol for PE diagnosis in the first trimester of pregnancy in the majority of women in this group. Moreover, V/P SPECT was demonstrated to be a safe procedure regarding sensitivity, negative predictive value and radiation exposure to the fetus and the pregnant woman. All reports were diagnostic. P SPECT alone was sufficient in 20 patients and identified women in whom it was necessary to perform the additional V SPECT to confirm or exclude PE and comorbidity. Furthermore, V SPECT helped to clarify the cause of symptoms by identifying additional diagnoses of pneumonia and chronic obstructive pulmonary disease (COPD). The majority of our patients thus benefited from V SPECT.

The prevalence of PE among pregnant women with suspected disease was highest during the first trimester, confirming results from other investigators [2]. However, the prevalence of PE in our study during the entire pregnancy was 9 %, which is higher than presented by others [16, 17]. The reason for this may be that previous investigators might have underestimated the true incidence either by not sending patients for imaging tests or alternatively by relying on MDCT which in the PIOPED II study [18] showed a comparably low sensitivity for PE diagnosis in the general population. Furthermore, Ridge et al. reported a high number of non-diagnostic findings on MDCT during pregnancy and therefore considered pulmonary planar scintigraphy more reliable [19]. Use of planar pulmonary scintigraphy as recommended by some guidelines [8, 9] is still often applied in many clinics although it is clearly less sensitive than V/P SPECT [20–23].

In this retrospective study, no V/P SPECT examination in pregnancy was not diagnostic, in contrast to the inconclusive results in 6 of the 61 (10%) women undergoing MDCT during the same period. Such a good performance is in line with other studies that have evaluated V/P SPECT for PE diagnosis in larger cohorts of non-pregnant patients [22, 24–27].

The debate regarding the preferred method of PE diagnosis in pregnancy is still ongoing. Guidelines in general recommend pulmonary scintigraphy as a first choice method [6, 8, 9]. However, American guidelines [9] recommend an MDCT if the chest X-ray is pathological. This is probably because planar scintigraphy traditionally has been used by the majority of institutions until now. The results of this study of pregnant patients and other studies in patients with suspected PE in general showed that a pathological X-ray is not a contraindication to the use of V/P SPECT. European guidelines, by contrast, recommend pulmonary SPECT as a first choice regardless of pathological chest X-ray [6].

In addition to sensitivity, one important reason supporting the use of V/P SPECT in pregnancy is safety, both for the fetus and the pregnant woman. The measured breast absorbed dose with V/P SPECT was 0.8 mGy in our study. This is a low figure in comparison to calculated breast absorbed doses of 20–50 mGy reported for MDCT [28–33]. Fetal absorbed doses, on the other hand, are similar for MDCT and V/P SPECT [29].

When choosing a diagnostic method, it is always important to assess its negative predictive value. None of the women in whom the P SPECT or V/P SPECT examination was negative for PE was diagnosed with PE or DVT later during the same pregnancy or the following puerperal period, confirming that the method performs well also in this respect. The V/P SPECT examination is thus a fast, safe and reliable way of excluding PE in this important patient group.

### Conclusion

This study showed that among pregnant women with suspected disease the incidence of a positive scan suggesting PE is low (9%), reaffirming the need for low radiation doses. The negative predictive value of V/P SPECT was high, and the radiation exposure from V/P SPECT was low, both for the fetus and the patient. A ventilation study was helpful to clarify patient symptoms and might be cost-effective regarding time and need for other examinations.
